# TEOSINTE BRANCHED1 regulates height and stem internode length in bread wheat

**DOI:** 10.1093/jxb/eraa252

**Published:** 2020-05-25

**Authors:** Laura E Dixon, Marianna Pasquariello, Scott A Boden

**Affiliations:** 1 Department of Crop Genetics, John Innes Centre, Colney Lane, Norwich, UK; 2 School of Biology, Faculty of Biological Sciences, University of Leeds, Leeds, UK; 3 School of Agriculture, Food and Wine, Waite Research Institute, Waite Research Precinct, University of Adelaide, Glen Osmond, SA, Australia; 4 University College Dublin, Ireland

**Keywords:** Architecture, coleoptile, growth, height, *TEOSINTE BRANCHED1* (*TB1*), wheat (*Triticum aestivum*)

## Abstract

Regulation of plant height and stem elongation has contributed significantly to improvement of cereal productivity by reducing lodging and improving distribution of assimilates to the inflorescence and grain. In wheat, genetic control of height has been largely contributed by the *Reduced height-1* alleles that confer gibberellin insensitivity; the beneficial effects of these alleles are associated with less favourable effects involving seedling emergence, grain quality, and inflorescence architecture that have driven new research investigating genetic variation of stem growth. Here, we show that TEOSINTE BRANCHED1 (TB1) regulates height of wheat, with *TB1* being expressed at low levels in nodes of the main culm prior to elongation, and increased dosage of *TB1* restricting elongation of stem internodes. The effect of *TB1* on stem growth is not accompanied by poor seedling emergence, as transgenic lines with increased activity of *TB1* form longer coleoptiles than null transgenic controls. Analysis of height in a multiparent mapping population also showed that allelic variation for *TB1* on the B genome influences height, with plants containing the variant *TB-B1b* allele being taller than those with the wild-type *TB-B1a* allele. Our results show that *TB1* restricts height and stem elongation in wheat, suggesting that variant alleles that alter the expression or function of *TB1* could be used as a new source of genetic diversity for optimizing architecture of wheat in breeding programmes.

## Teaser

Increased dosage of *TB1* reduces height and stem internode length in bread wheat without affecting coleoptile length.

## Introduction

Height is an important trait in cereals including wheat, rice, and barley, with genetic variation that reduces stem elongation having contributed significantly to the generation of superior yielding cultivars during the ‘Green Revolution’ (Hedden, 2003). Plants with reduced height benefit from improved lodging resistance, and the suppression of growth increases the ability of plants to tolerate higher inputs of nitrogen-based fertilizer; the decrease in stem elongation boosts partitioning of assimilates to the developing inflorescence, facilitating improved floret fertility and grain numbers per ear (Youssefian *et al.*, 1992*a*; Flintham *et al.*, 1997)

Genetic variation that contributes semi-dwarfing alleles in wheat, barley, and rice mostly involves modification of biosynthesis, metabolism. or signalling of the growth-promoting phytohormone, gibberellic acid (GA) (Hedden, 2003; Pearce *et al.*, 2011). For example, the semi-dwarfing *Reduced height-1* (*Rht-1*; *Rht-B1b* and *Rht-D1b*) alleles encode variants of the DELLA protein that no longer confer responsiveness to GA, with the DELLA protein being a negative regulator of GA signalling that represses growth (Peng *et al.*, 1999; de Lucas *et al.*, 2008; Feng *et al.*, 2008; Pearce *et al.*, 2011). In rice and barley, the semi-dwarfing *sd1* and *sdw1* alleles contain loss-of-function mutations in the key GA biosynthesis enzyme GA 20-oxidase 2 (GA20ox2)—the defective alleles reduce levels of the growth-promoting bioactive GAs (Spielmeyer *et al.*, 2002; Jia *et al.*, 2009; Xu *et al.*, 2017). In addition to the beneficial height reduction effects of the semi-dwarfing *Rht-1* alleles, the decreased responsiveness to GA provokes unfavourable pleiotropic phenotypes including reduced seedling emergence, lower photosynthetic rate, and decreased grain size and grain protein content, which has encouraged research of novel *Rht-B1* alleles that contain second site mutations and other dwarfing alleles, such as *Rht8* and *Rht18/Rht25* (Allan, 1980; Youssefian et al., 1992*a*, *b*; Schillinger *et al.*, 1998; Rebetzke *et al.*, 1999; Wu *et al.*, 2011; Gasperini *et al.*, 2012; Chandler and Harding, 2013; Casebow *et al.*, 2016; Kowalski *et al.*, 2016; Van De Velde *et al.*, 2017; Ford *et al.*, 2018; Mo *et al.*, 2018; Jobson *et al.*, 2019). *Rht18* encodes a GA 2-oxidase, which is responsible for converting bioactive GA into inactive forms of the hormone (Ford *et al.*, 2018). The effect of these *Rht* alleles on stem elongation and height is accompanied by changes in physical characteristics of the inflorescence; dwarfed plants develop compact spikes with an altered number of spikelets (Mo *et al.*, 2018).

Inflorescence development is closely associated with stem elongation, as elongation of stem internodes 2 and 3 occurs simultaneously with the white anther and terminal spikelet stages, respectively, which are when floret and spikelet numbers are determined (Kirby, 1987; Reynolds *et al.*, 2009; Guo and Schnurbusch, 2015). To identify genes that influence spikelet and floret formation, we and others have studied genes that alter the rate of inflorescence development, spikelet architecture, and/or floret fertility (Boden *et al.*, 2015; Guo and Schnurbusch, 2015; Dixon *et al.*, 2018; Sakuma *et al.*, 2019). We showed that a wheat orthologue of the major maize domestication gene, *TEOSINTE BRANCHED1* (*TB1*), is a key regulator of plant architecture and inflorescence development (Dixon *et al.*, 2018). Increased dosage of *TB-D1* (D genome homeologue of *TB1*) delayed inflorescence development, restricted tiller growth, and promoted formation of paired spikelets, which are supernumerary spikelets characterized by the development of two spikelets at a single rachis node, rather than the typical single spikelet (Boden *et al.*, 2015). These traits are all linked physiologically by the suppression of growth, particularly of axillary meristems including spikelet primordia and tiller buds, with a decrease in the rate of inflorescence development occurring at the terminal spikelet stage associated with elongation of stem internode 3 (Dixon *et al.*, 2018). The effect of *TB1* on growth is consistent with it being a class II TCP transcription factor, which regulates expression of genes involved in processes including cell proliferation, the cell cycle, as well as signalling and metabolism of hormones such as GA and jasmonic acid; TB1 and its Arabidopsis homologue, BRANCHED1 (BRC1), inhibit expression of cell cycle genes and those involved in GA signalling (González-Grandío *et al.*, 2013; Dong *et al.*, 2019). Based on the role of TB1/BRC1 controlling cell proliferation, and its key role in regulating the rate of inflorescence development and plant architecture, we hypothesized that *TB1* may influence the height of wheat plants.

In this study, we investigated height and stem internode lengths in wheat lines that contain increased dosage of *TB-D1*, including a pair of near-isogenic lines (NILs) developed from a MAGIC population that are tetrasomic for chromosome 4D and transgenic plants that express *TB1* at higher levels (Dixon *et al.*, 2018). We show that increased activity of *TB1* restricts height and stem elongation in bread wheat, and that allelic diversity for *TB-B1* influences height in an advanced mapping population. These results provide new insights into the genetic regulation of height in wheat, which may help breeders optimize plant architecture.

## Materials and methods

### Plant materials and growth conditions

Hexaploid wheat (*Triticum aestivum*) germplasm used in this study included the following genotypes: MAGIC line 0053 of the CSIRO four-way multiparent advanced generation intercross (MAGIC) population, from which the NILs termed wild type (WT) and *highly-branched* (*hb*) were derived (see Dixon *et al.*, 2018); transgenic lines expressing *TB-D1* using the *VRN1* promoter generated in the cv. Fielder genetic background (Dixon *et al.*, 2018); and the hexaploid wheat TILLING line, *Cadenza1721* (*tb-d1*) (Krasileva *et al.*, 2017). The *hb* line is tetrasomic for chromosome 4D and contains an additional haploid copy of *TB-D1*, relative to its WT NIL that is diploid for 4D (see Dixon *et al.*, 2018). The *pVRN1:TB1* transgenic lines expressed the *TB-D1* allele from cv. Baxter that is present in *hb* and WT NILs of the MAGIC line 0053, under the control of the barley *VRN1* promoter (Alonso-Peral *et al.*, 2011). The ‘NIAB Elite MAGIC’ bread wheat population has been previously described (Mackay *et al.*, 2014), and height data were collated from published data of plants grown under field conditions at the NIAB experimental farm in Cambridge, UK (52°13'19''N, 0°5'46''E) (Mackay *et al.*, 2014; Scutari *et al.*, 2014).

WT, *hb* plants, transgenic lines, and the F_1_ plants of the *hb* crosses to Cadenza, *tb-d1* (*Cadenza1721*; see Supplementary Fig. S1 at *JXB* online), that were used for phenotype analysis and gene expression studies were grown in controlled growth chambers under long-day (16 h light/8 h dark) photoperiods at 300 µmol m^–2^ s^–1^ [using Plantastar 400-W HQI bulbs (Osram) and Maxim 60 W tungsten bulbs], with a day temperature of 20 °C and a night temperature of 15 °C.

### RNA extractions and quantitative real-time PCR analysis

For experiments with WT and *hb* plants, RNA was extracted from internode 2 stem, prior to internode elongation. For experiments using the *pVRN1:TB1* transgenic and null control lines, including cv. Fielder, RNA was extracted from internode 2 stem, prior to initiation of elongation. For transcript analysis of *TB-B1* and *TB-D1* in cv. Cadenza, RNA was extracted from internodes 1, 2, and 3 and the peduncle prior to initiation of elongation and from tiller buds, which are immature tillers that were transparent or light green in colour for which no leaf blade had begun to emerge. All RNA extractions were performed using the RNeasy Plant Mini Kit (Qiagen). Synthesis of cDNA and quantitative real-time PCR (qRT-PCR) were performed as described previously (Boden *et al.*, 2014). Oligonucleotides for qRT-PCR analysis are provided in Supplementary Table S1. Expression of *TB-B1* and *TB-D1* was normalized using Traes_6DS_BE8B5E56D.1 (gene ID in IWGSC reference genome is TraesCS6D02G145100) (Borrill *et al.*, 2016). All qRT-PCR data points are the average of at least four biological replicates, with two technical replicates performed in each reaction.

### Analysis of coleoptile elongation

Coleoptile length measurements were performed using seeds of equal weight (between 40 mg and 50 mg), with individual seeds sown into pots (dimensions 90×90×180 mm) containing fertile compost-based potting mix at a sowing depth of ~5 cm. The soil was initially watered until saturation (~150 ml), and pots were placed in growth chambers and covered with lids wrapped in aluminium foil. Lights were turned off and the temperature was set to 20 °C/15 °C (day/night). Pots were watered equally every 2 d (15 ml). Coleoptile length was measured using a ruler, 12 d after sowing once the cotyledon had emerged from the coleoptile. Each experiment was performed at least three times, with 12 seeds per genotype per replicate.

### Kompetitive allele-specific PCR analysis

Kompetitive allele-specific PCR (KASP-PCR) analysis was performed as per Dixon *et al.* (2018). Sequences for oligonucleotides used in KASP assays are provided in Supplementary Table S2.

### Statistical analysis

Differences between genotypes were tested by two-tailed Student’s *t*-test. The number of replicates for each experiment are provided elsewhere in the Materials and methods and in the relevant figure legend.

### Accession numbers

The gene IDs for *TB-A1*, *TB-B1*, and *TB-D1* are TraesCS4A02G271300, TraesCS4B02G042700, and TraesCS4D02G040100, respectively.

## Results

### Increased dosage of *TB-D1* reduces height and internode length in bread wheat

**Fig. 1.**
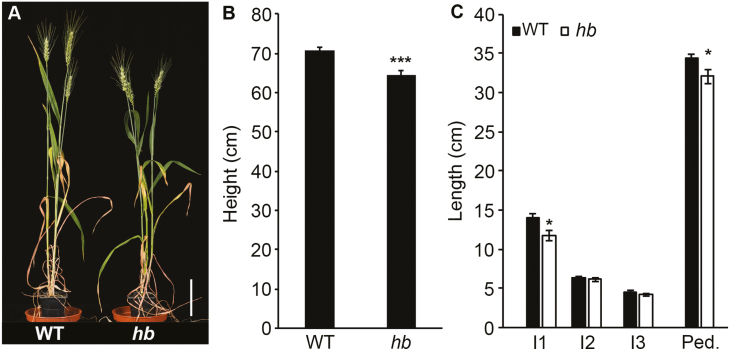
Tetrasomy for chromosome 4D reduces plant height. (A and B) *hb* plants that are tetrasomic for chromosome 4D are shorter than wild-type near-isogenic lines. (C) Internode lengths for wild-type and *hb* plants. I1, internode 1; I2, internode 2; I3, internode 3; Ped., peduncle. Scale bar=10 cm. Values are the mean ±SE of 10 biological replicates. *P<0.05; ***P<0.001.

**Fig. 2.**
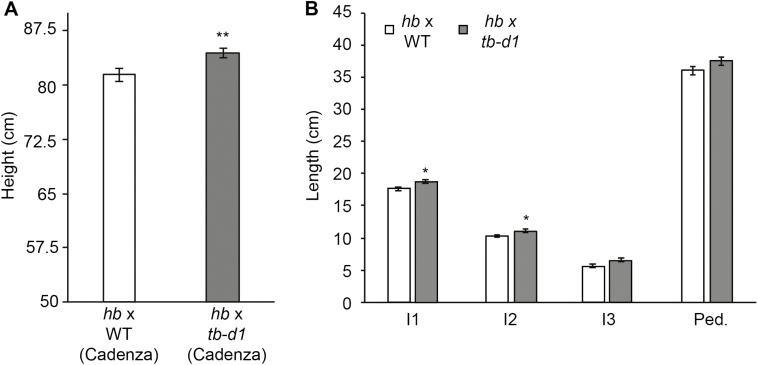
*TB-D1* contributes to the reduced height of *hb* plants. (A) Height and (B) internode length measurements of F_1_ offspring plants from crosses between *hb* and Cadenza (WT), and *hb* and the *tb-d1* null mutant line (*Cadenza1721*). I1, internode 1; I2, internode 2; I3, internode 3; Ped., peduncle. Values are the mean ±SE of 48 biological replicates. *P<0.05; **P<0.01.

Our previous investigation of inflorescence and plant architecture included analysis of a pair of NILs derived from a single line of a four-way MAGIC population (Dixon *et al.*, 2018). The NIL *hb* formed multiple paired spikelets on the inflorescence, relative to the WT NIL that formed an inflorescence with single spikelets at each rachis node. The *hb* line is tetrasomic for chromosome 4D, relative to the euploid WT line. In addition to the inflorescence and tiller phenotypes, the *hb* line was shorter than the WT. To investigate this trait further and determine if *TB1* influences stem growth, we measured height and internode length in *hb* plants grown under long-day photoperiods, relative to the WT. The *hb* plants were significantly shorter than the WT (Fig. 1A, B), and lengths of internode 1 and the peduncle were significantly shorter in *hb* compared with the WT, while there was no significant difference in the length of internodes 2 and 3. This result demonstrates that increased dosage of chromosome 4D restricts plant height in wheat. To test if this effect of tetrasomy 4D is caused by increased dosage of *TB1* and not another gene on chromosome 4D, we crossed the *hb* line to a Cadenza TILLING mutant that contains a premature stop codon in *TB-D1* (*tb-d1*) and analysed height and internode length of the first filial generation (F_1_) individuals, relative to F_1_ individuals from a cross between *hb* and Cadenza (Cadenza contains WT tall alleles of *Rht-B1* and -*D1*). We observed that the F_1_ individuals of the *hb*×*tb-d1* individuals were taller than those derived from the *hb*×Cadenza cross (Fig. 2A). The lengths of internodes 1 and 2 were longer in the *hb*×*tb-d1* F_1_ individuals, relative to individuals of the *hb*×Cadenza cross, while there was no significant difference in the length of internode 3 (Fig. 2B). Taken together, these results indicate that tetrasomy for chromosome 4D restricts plant height and internode length in wheat, and that increased dosage of *TB-D1* contributes to this effect on growth.

### Expression analysis of *TB-1* in stem tissue

**Fig. 3.**
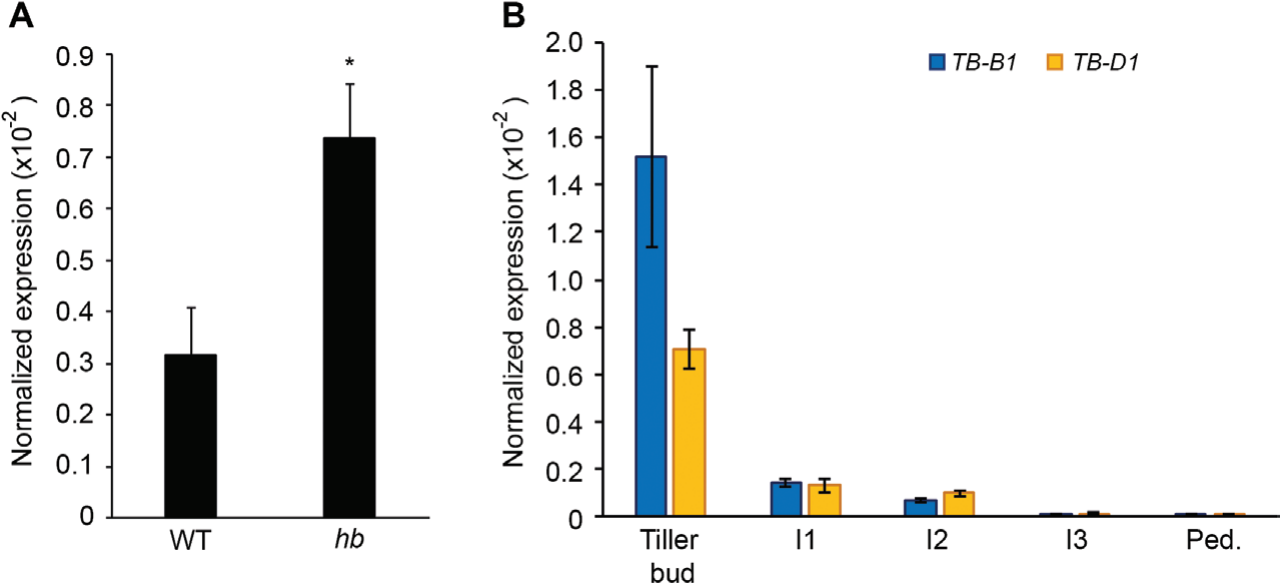
*TB-B1* and *TB-D1* expression in stem tissue. (A) Transcript analysis of *TB-D1* in internode 2 stem tissue of WT and *hb* lines. (B) Quantification of *TB-B1* (blue) and *TB-D1* (yellow) transcripts in stem tissue and tiller buds. I1, internode 1; I2, internode 2; I3, internode 3; Ped., peduncle. Values are the mean ±SE of four biological replicates. *P<0.05.

To determine if the effect of *TB-D1* on height and internode length in the *hb* plants could be explained by increased expression of *TB-D1*, we measured *TB-D1* transcript levels in internode 2 stem regions prior to elongation. qRT-PCR analysis detected *TB-D1* transcripts in the stem internode 2 in both WT and *hb* plants, with transcript levels being ~2-fold higher in *hb* relative to the WT (Fig. 3A). Transcript analysis in Cadenza showed that *TB-D1* and *TB-B1* are expressed in internodes 1 and 2 prior to elongation of the internodes, and the levels are low relative to those measured in tiller buds which we demonstrated previously to express *TB1* (Fig. 3B); transcript levels in Cadenza were slightly lower than those detected in the same internode of the WT NIL. We did not measure *TB-A1* expression because our previous analysis has shown that this homoeoallele is not expressed (Dixon *et al.*, 2018). These results show that reduced height and internode lengths of the *hb* plants are associated with increased expression of *TB-D1* in stem internode tissue prior to elongation, and that *TB1* is expressed at low levels in stem segments prior to elongation of the internode.

### Analysis of height in *pVRN1:TB-D1* transgenic lines

**Fig. 4.**
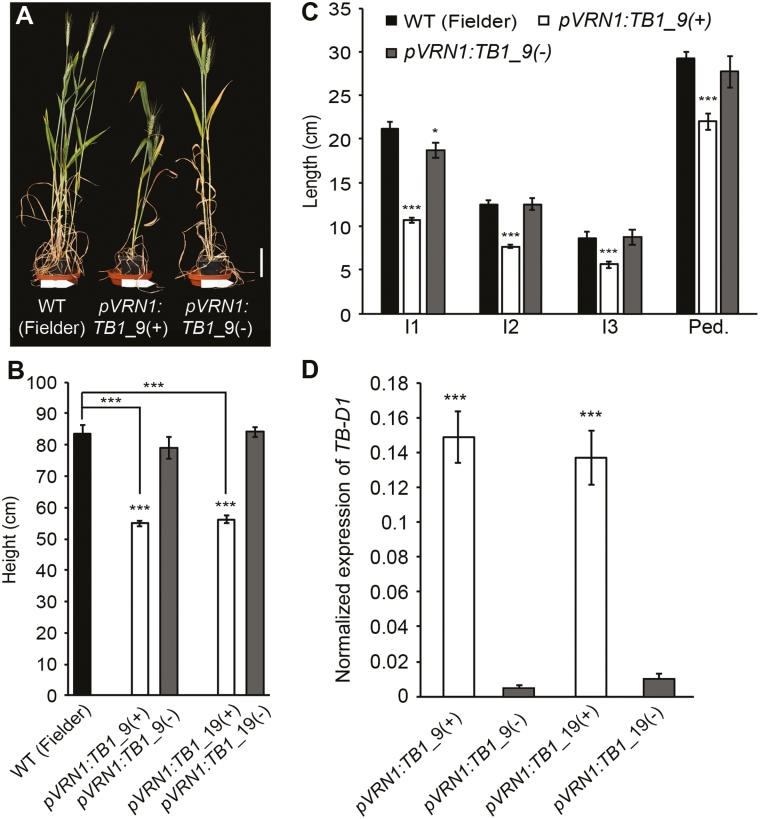
Increased dosage of *TB-D1* reduces plant height. (A and B) Height and (C) internode length measurements for two independent *pVRN1:TB1* transgenic lines (white bars), relative to control null transgenic lines (grey bars) and the untransformed wild type (cv. Fielder, black bars). (D) *TB-D1* expression in internode 2 of the elongating stem of *pVRN1:TB1* transgenic plants (white bars), relative to control null transgenic lines (grey bars). I1, internode 1; I2, internode 2; I3, internode 3; Ped., peduncle. Scale bar=10 cm. (A–C) Values are the mean ±SE of 10 biological replicates, and (D) the mean ±SE of five biological replicates. *P<0.05; ***P<0.001.

To further test if increased dosage of *TB-D1* restricts stem growth and height in wheat, we analysed transgenic lines that contain an additional copy of *TB-D1* under control of the *VERNALIZATION1* (*VRN1*) promoter (*pVRN1:TB1*) (Alonso-Peral *et al.*, 2011). The *pVRN1:TB1* transgenic lines were significantly shorter than control null transgenic lines [*pVRN1:TB1*(–)] and the untransformed plants (cv. Fielder) (Fig. 4A, B). The *pVRN1:TB1* transgenic lines had shorter internodes than the null transgenic control lines and cv. Fielder, with length of internodes 1, 2, and 3 and the peduncle being significantly reduced, relative to the control lines (Fig. 4C). The stem growth and height phenotypes are associated with significantly higher transcript levels of *TB-D1* in stem segments prior to stem elongation, relative to the null transgenic lines (Fig. 4D). Increased expression of *TB-D1* is consistent with *VRN1* transcript levels being relatively high in stem tissue compared with leaf tissue where it is expressed robustly (Supplementary Fig. S2). These results show that increased expression of *TB-D1* within developing stem tissue restricts internode elongation and plant height in wheat, which support the phenotypes of the *hb* line relative to the WT NIL.

### Increased dosage of *TB-D1* does not restrict coleoptile length

**Fig. 5.**
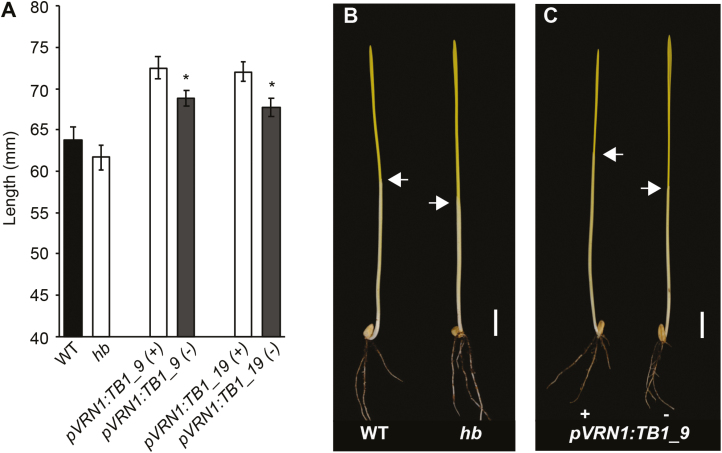
Analysis of the effect of *TB-D1* on coleoptile length. (A) Coleoptile length measurements for WT (black) and *hb* (white) lines, and for *pVRN1:TB-D1* transgenic lines (grey) relative to the null transgenic control lines (white). Images of representative seedlings for (B) WT and *hb* and for (C) transgenic *pVRN1:TB-D1* transgenic and null control lines. Scale bar=1 cm; white arrows indicate the apical end of the coleoptile. Values are the mean ±SE for 12 biological replicates. * *P*<0.05.

A limitation of using *Rht-b1b* and *Rht-D1b* dwarfing alleles in modern wheat is that the benefit of reduced stem elongation to reduce lodging is accompanied by unfavourable pleiotropic traits including poor emergence of seedlings from deep sowing, decreased meristem size, lower grain set, increased susceptibility to diseases, and reduced 1000-grain weight (Allan, 1980; Borner *et al.*, 1996; Schillinger *et al.*, 1998; Rebetzke *et al.*, 1999; Ellis *et al.*, 2004; Saville *et al.*, 2012; Serrano-Mislata *et al.*, 2017). We have shown that increased dosage of *TB1* alters spikelet architecture and delays stages of inflorescence development critical for floret formation (Dixon *et al.*, 2018); here, we tested if increased dosage of *TB1* reduced coleoptile length to determine if it would restrict seedling emergence from soil. We found that the coleoptile length of *hb* plants was shorter than that of the WT, but was longer in two independent *pVRN:TB1* transgenic lines, relative to null transgenic plants (Fig. 5A–C). These results show that tetrasomy for chromosome 4D reduces coleoptile length, which is consistent with increased dosage of *Rht-D1a* (also located on 4D); however, increased dosage of *TB1* alone does not restrict coleoptile growth or emergence of seedlings. These results indicate that increased activity of *TB1* restricts plant height without negatively influencing seedling emergence, suggesting that alleles with increased activity of *TB1* could be used as an alternative to *Rht-B1b* or *Rht-D1b* semi-dwarfing alleles.

### Allelic variation for *TB-B1* influences plant height in bread wheat

**Fig. 6.**
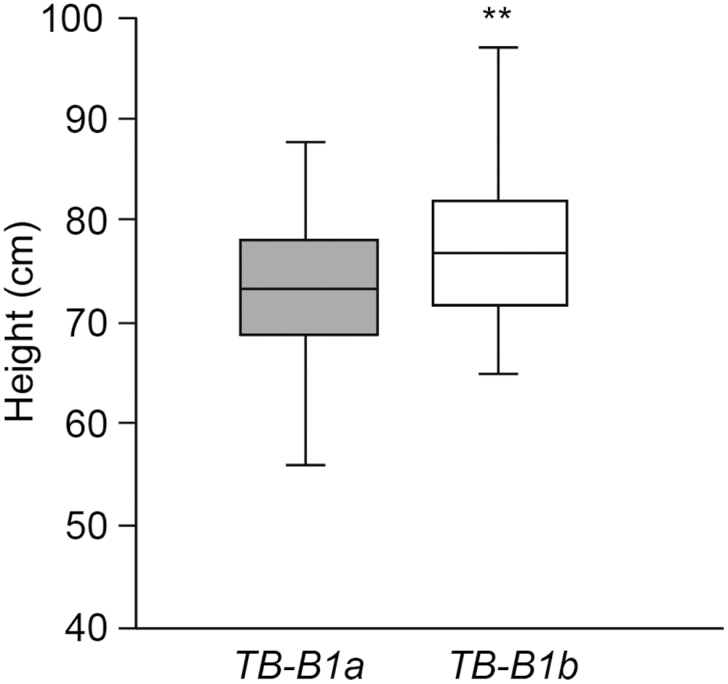
Analysis of allelic variation for TB-B1 on height phenotypes. Boxplots showing the height of semi-dwarfed eight-way MAGIC lines (genotype *Rht-D1b*) that contain either the *TB-B1a* (grey) or the *TB-B1b* (white) allele. Boxes extend from the lower to the upper quartile, with a line marking the median, and whiskers extend to 1.5 times the interquartile range. **P<0.01.

Our previous analysis of *TB1*-dependent regulation of inflorescence architecture identified a variant allele for *TB-B1* (known as *TB-B1b*), which is predicted to be a weak loss-of-function allele that contains three amino acid substitutions and two synonymous mutations, relative to the reference allele (Dixon *et al.*, 2018). Based on our results showing that increased activity of *TB-D1* reduces height (Figs. 1, 4), and *TB-B1* being expressed equally to *TB-D1* (Fig. 3), we hypothesized that the *TB-B1b* allele would increase height of bread wheat, relative to the *TB-B1a* allele. To test this hypothesis, we analysed the height of lines from the eight-way UK MAGIC population that were fixed for the semi-dwarfing allele of *Rht-D1* (*Rht-D1b*; aka *Rht-2*) but differed with respect to the *TB-B1* allele (i.e. *Rht-D1b*:*TB-B1a*, or *Rht-D1b*:*TB-B1b*) (Scutari *et al.*, 2014; Mackay *et al.*, 2014). We did not analyse any lines with two dwarfing alleles (*Rht-B1b*/*Rht-D1b*) or any of the *Rht-B1b* semi-dwarf lines because they all contained the *TB-B1a* allele, which is consistent with *Rht-B1* and *TB-B1* being in close genetic linkage with each other (Dixon *et al.*, 2018). We found that lines containing the *TB-B1b* allele were significantly taller than lines with the *TB-B1a* allele (Fig. 6), with *TB-B1b* genotypes being 4.6% taller (3.4±0.91 cm; *P*<0.01) than *TB-B1a* genotypes. These results support the hypothesis that *TB-B1b* alleles are weak loss-of-function alleles that can partially restore the height of lines that contain dwarfing alleles of *Rht-1.*

## Discussion

The genetic regulation of height has contributed significantly to improvement of crop productivity, especially in wheat, rice, and barley, by reducing stem elongation to improve lodging resistance and enhance partitioning of resources to the inflorescence (Hedden, 2003). In wheat, these benefits have largely been provided by the semi-dwarfing *Rht-B1b* and *Rht-D1b* alleles (Peng *et al.*, 1999; Pearce *et al.*, 2011); however, due to their pleiotropic effects on seedling emergence, spikelet number, grain weight, and quality (for example), research has continued to investigate novel sources of genetic variation for control of height (Allan, 1980; Youssefian et al., 1992*a*, *b*; Schillinger *et al.*, 1998; Rebetzke *et al.*, 1999; Wu *et al.*, 2011; Gasperini *et al.*, 2012; Kowalski *et al.*, 2016; Ford *et al.*, 2018; Mo *et al.*, 2018). Here, we have shown that increased expression of *TB1* reduces stem elongation in bread wheat, and that allelic variation for *TB-B1* influences plant height.

*TB1* and its homologue in eudicots—BRANCHED1 (*BRC1*)—have been studied extensively due to their role in regulating shoot branching and apical dominance (Doebley *et al.*, 1997; Aguilar-Martínez *et al.*, 2007). *TB1*/*BRC1* of maize, rice, wheat, barley, tomato, pea, Arabidopsis, switchgrass, and cucumber have conserved roles in regulating outgrowth of lateral branches, with loss-of-function mutations increasing lateral branch number and length, while gain-of-function alleles suppress their growth (Doebley *et al.*, 1997; Takeda *et al.*, 2003; Aguilar-Martínez *et al.*, 2007; Weber *et al.*, 2007; Minakuchi *et al.*, 2010; Martín-Trillo *et al.*, 2011; Ramsay *et al.*, 2011; Braun *et al.*, 2012; Seale *et al.*, 2017; Bennett *et al.*, 2016; Dixon *et al.*, 2018; Liu *et al.*, 2018; Zwirek *et al.*, 2019; Shen *et al.*, 2019). A conserved role for *TB1*/*BRC1* in controlling height and stem elongation is, however, less clear; for example, *brc1* and *brc1/brc2* Arabidopsis mutants are shorter than the WT, as are tomato plants with reduced expression of *BRC1*, potentially due to reduced apical dominance (Finlayson *et al.*, 2010; Martín-Trillo *et al.*, 2011; Bennett *et al.*, 2016; Seale *et al.*, 2017). The effect of *tb1/brc1* loss-of-function alleles in eudicots is maintained in rice, with loss-of-function mutants of *TB1*, known as *FINE CULM1* (*FC1*), shorter than WT plants (Minakuchi *et al.*, 2010). Analysis in maize did not detect a role for allelic diversity of *Tb1* in controlling height, and there is no statistical difference in height for barley and switchgrass lines that contain alleles of reduced *TB1/VRS5* expression (Liu *et al.*, 2018; Zwirek *et al.*, 2019). These reports seemingly show that wheat differs from other plants, as our results show that increased expression of *TB1* reduces height, and that alleles with reduced function are likely to partially restore height in semi-dwarfed plants (Figs. 1, 4, 6). However, research in model and crop species has mostly involved analysis of reduced function alleles, relative to the gain-of-function alleles tested here. This conclusion is supported by reports showing that overexpression of the maize *Tb1* gene in wheat reduces plant height, and induced expression of *BRC1* leads to severe arrested growth of Arabidopsis seedlings (Lewis *et al.*, 2008; González-Grandío *et al.*, 2013). The results presented here suggest that further investigation of *TB1* variant alleles, particularly those that influence expression through gene editing of *cis*-regulatory regions, may prove to be useful in regulating stem elongation and height.

Analyses of *TB1* in wheat have demonstrated roles for this gene in regulating inflorescence development and plant architecture, including height and tillering (Dixon *et al.*, 2018). A link between inflorescence architecture genes and height has also been shown in other crops. For example, in barley, a major inflorescence trait is row-type architecture, with cultivated barley forming spikes with either two or six row arrangements of spikelets; this trait is regulated by at least five genes, *VRS1– VRS5* (*VRS5* is a *TB1* homologue) (reviewed in Gauley and Boden, 2019). Analysis of plant architecture in the *vrs* mutants has shown that some of the *VRS* row-type alleles influence plant height—*vrs4* and *vrs4/5* mutants are significantly taller than WT (cv. Bowman) plants, while *vrs1/5* and *vrs3/5* double mutants are significantly shorter (Zwirek *et al.*, 2019). Moreover, barley *deficiens* mutants that maintain protein stability of *VRS1* display reduced plant height, relative to NILs with WT *VRS1* alleles (Sakuma *et al.*, 2017). In domesticated wheat, a mutation in the miRNA172- (miR172) binding site of an AP2-like transcription factor that underpins the *Q* locus have contributed to development of a compact spike and a free-threshing phenotype, relative to the elongated rachis of speltoid wheat (Debernardi *et al.*, 2017; Greenwood *et al.*, 2017). Further mutations that disrupt binding of miR172 modify spike compactness further, and are associated with significant reduction in plant height (Debernardi *et al.*, 2017; Greenwood *et al.*, 2017). Inflorescence architecture and height are also associated in rice; for example, the *SQUAMOSA PROMOTER BINDING-LIKE PROTEIN-14* (*SPL14*) gene that underpins the *IDEAL PLANT ARCHITECTURE* (*IPA*)/*WEALTHY FARMERS PANICLE* (*WFP*) locus influences panicle branching and plant height (Jiao *et al.*, 2010; Miura *et al.*, 2010). Mutations in a miR156-binding site increase expression of *SPL14* to promote formation of a more highly branched panicle with improved lodging resistance and reduced tillering. Taken together, these results demonstrate an important link between the genetic regulation of inflorescence and plant architecture, and highlight the importance of considering pleiotropic effects of genes when determining genetic variation that may be used to improve crop performance.

The influence of *TB1* on height in wheat, including our analysis suggesting that the variant *TB-B1b* allele partially restores stem length in semi-dwarfed germplasm, provides an opportunity to investigate genetic variation that will help improve plant architecture in breeding programmes (Fig. 6). Analysis of *Rht-B1b* and *Rht-D1b* alleles has shown that semi-dwarfism is associated with unfavourable pleiotropic traits, including reduced seedling emergence from deep sowing, decreased grain size, and lower grain protein content, which has stimulated investigation of alternative *Rht* alleles, such as *Rht8*, *Rht12*, and *Rht18/25* (Youssefian et al., 1992*a*, *b*; Rebetzke *et al.*, 1999; Gasperini *et al.*, 2012; Kowalski *et al.*, 2016; Mo *et al.*, 2018). *Rht8* is particularly interesting as it is likely to encode a protein that is independent of the GA metabolism and signalling pathways, and does not restrict coleoptile growth (Rebetzke *et al.*, 1999; Gasperini *et al.*, 2012). Our results suggest that increased expression of *TB1* has a similar effect—elevated activity of *TB1* reduced height and stem internode length without negatively affecting coleoptile growth (Figs 4, 5). Alleles that increase expression of *TB1* may therefore be useful in restricting stem elongation and tillering without decreasing seedling emergence, providing an alternative to other *Rht* alleles. The *Rht-B1b* and *Rht-D1b* alleles reduce height to ~86% and 83% of tall controls, respectively, and *Rht8* dwarfing alleles decrease height by 11%, relative to tall NILs (Flintham *et al.*, 1997; Kowalski *et al.*, 2016). Based on the results shown here, alleles that increase expression of *TB1* have potential to reduce height similarly to *Rht8* (91% of the height of WT controls) in tetrasomic plants, while the more highly expressed *pVRN1:TB1* transgenes decreased height by ~30% (Figs 1, 4). Alleles that increase expression of *TB-B1* or *-D1* may therefore benefit breeding programmes by providing a comparable reduction in height to existing *Rht* alleles. Alternatively, *TB1* alleles that reduce functionality may also benefit wheat breeding. For example, the effect of TB1 on tillering involves hormones such as abscisic acid, jasmonic acid, and GA, indicating that TB1-dependent regulation of growth is at least partially separable from the molecular role of *Rht1* (Peng *et al.*, 1999; de Lucas *et al.*, 2008; Feng *et al.*, 2008; Dong *et al.*, 2019). The *TB-B1b* allele, or variant *TB-D1b* allele, could be introduced into *Rht-B1b* and *Rht-D1b* semi-dwarfed backgrounds to help fine-tune plant architecture traits including height, tillering, and spikelet number for improved crop performance (Dixon *et al.*, 2018). Due to the genetic linkage of *TB1* and *Rht1* on chromosome group 4, the most efficient breeding strategy would most probably involve combining reciprocal alleles on the B and D genomes, to form genotypes such as *Rht-D1b*:*TB-B1b* or *Rht-B1b:TB-D1b.* Further investigation of variant *TB1* alleles in different semi-dwarfed backgrounds will be necessary to determine the optimal allelic combinations for improved plant architecture.

In summary, our results demonstrate a new role for *TB1* in wheat, where increased activity reduces internode elongation and height (Figs. 1, 2, 4). Together with our previous report showing that *TB1* controls inflorescence development and tillering, these results show that *TB1* is an important regulator of plant architecture—further investigation of *TB1* will be critical for identifying pathways that act downstream to regulate tissue-specific phenotypes, which may provide opportunities to optimize key yield-related traits including tillering, spikelet number, and height.

## Supplementary data

Supplementary data are available at *JXB* online.

Fig. S1. Schematic of the *hb*×*tb-d1* cross and the control *hb*×Cadenza cross.

Fig. S2. Tissue-specific analysis of *VRN1* expression in wild-type wheat (cv. Cadenza)

Table S1. Oligonucleotide sequences used for qRT-PCR assays.

Table S2. Oligonucleotide sequences used for KASP-PCR assays

Table S3. *TB-B1* genotype information of MAGIC elite lines used to investigate height.

eraa252_suppl_Supplementary_Figures_S1-S2_Tables-S1-S3
