# Supplementary material for: TEOSINTE BRANCHED1 regulates height and stem internode length in bread wheat
Source: J Exp Bot. 2020 May 25;71(16):4742–50. doi: 10.1093/jxb/eraa252 (PMC7410180; doi:10.1093/jxb/eraa252)
Supplement: eraa252_suppl_Supplementary_Figures_S1-S2_Tables-S1-S3 [file eraa252_suppl_supplementary_figures_s1-s2_tables-s1-s3.pdf]

Supplementary Data

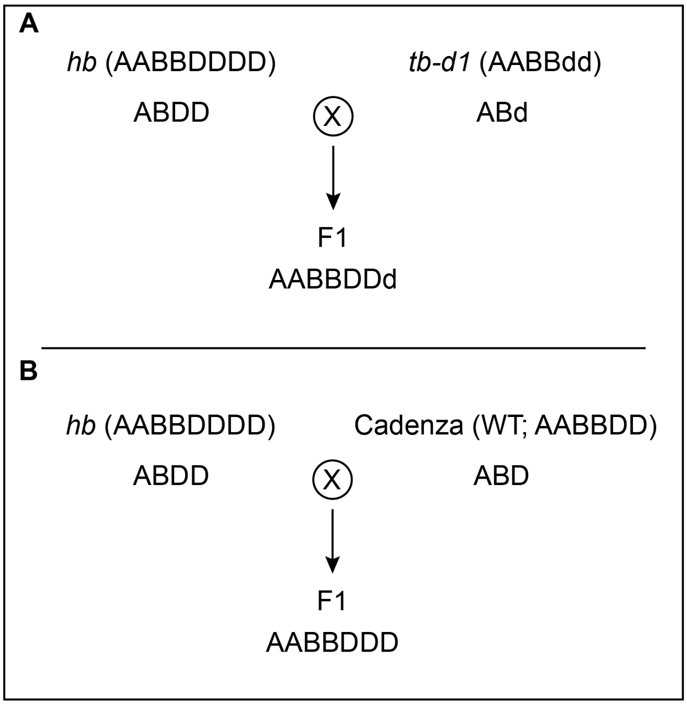

Supplementary Figure S1. Schematic of the *hb* × *tb-d1* cross, and the control hb × Cadenza cross. Schematic diagrams showing the crossing strategy of the *hb* line with either **(A)** *tb-d1* stop codon mutant (*Cad1721*) or **(B)** wild-type (Cadenza). A, B and D refer to *TB-A1*, *TB-B1* and *TB-D1* respectively, with '*d'* referring to the nonsense allele of *TB-D1*. The diploid genotype is shown in brackets, and the gamete genotype is shown below. The F1 genotype is shown for the respective crosses.

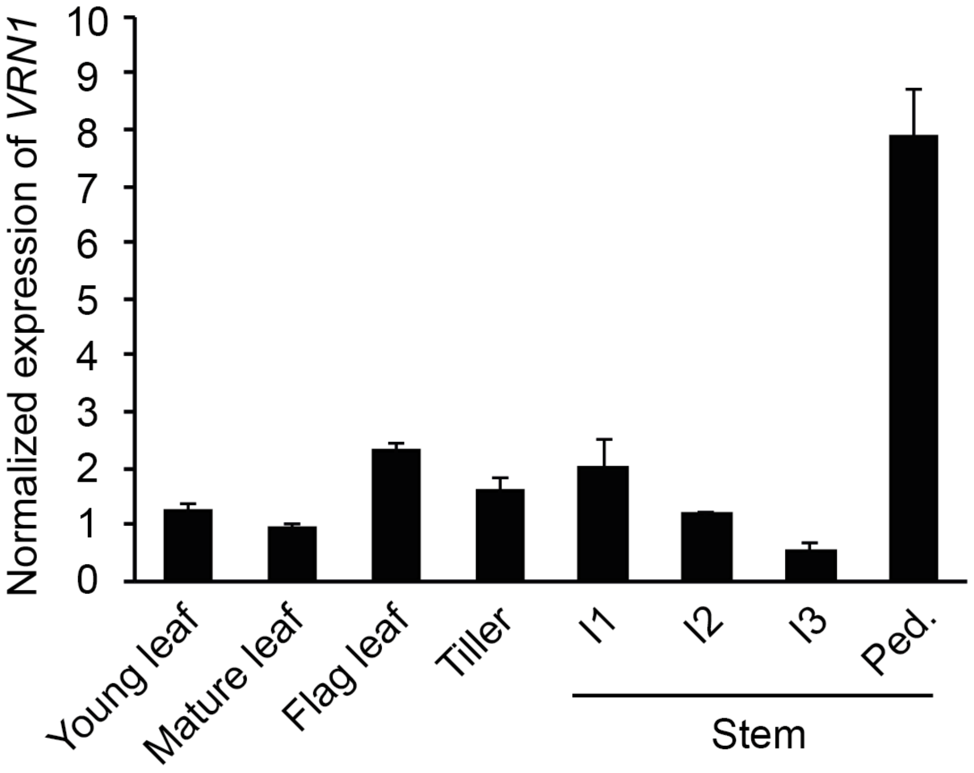

Supplementary Figure S2. ***VRN1*** **tissue specific expression analysis.** Transcript analysis of *VRN1* in leaf, tiller and stem tissue. I1, internode 1; I2, internode 2; I3, internode 3; Ped., peduncle. Values are mean ± s.e.m of 4 biological replicates.

Supplementary Table S1. Oligonucleotide sequences used in qRT-PCR assays.

Gene	Gene ID	Forward (sense) sequence	Reverse (anti-sense) sequence
TB-B1	TraesCS4B02G042700	ACCCGCCAAACCAAAGC	CATCCGGTTCTTTTCCCTAGT
TB-D1	TraesCS4D02G040100	CACCCGCCAATCCAAAAC	ATCCGGTTCTTTTCCCTCGT
Traes_6DS_BE8B5E56D.1	TraesCS6D02G145100	CATGCTCTGGGATTTATCCAT	CTGGATCATTTCCGGTGC
TaRPA-15		GCACACGTGCTTTGCAGATAAG	GCCCTCAAGCTCAACCATAACT

Supplementary Table S2. Oligonucleotide sequences used in KASP marker assays.

Gene	WT allele oligonucleotide (FAM)	Alternate allele oligonucleotide (HEX)	Common allele	Source
TB-B1	GAAGGTGACCAAGTTCATGCT-TTCCTTTGCGGCTTTGGT	GAAGGTCGGAGTCAACGGATT-TTCCTTTGCGGCTTTGGA	CGGCGAGGGGAAGAAGCA	Dixon et al. 2018
Rht-B1	GAAGGTGACCAAGTTCATGCT-CCCATGGCCATCTCCAGCTG	GAAGGTCGGAGTCAACGGATT-CCCATGGCCATCTCTAGCTA	TCGGGTACAAGGTGCGGGCG	Simon Griffiths, JIC
Rht-D1	GAAGGTGACCAAGTTCATGCT-CGTGGCGCAGAAGCTGG	GAAGGTCGGAGTCAACGGATT-CGTGGCGCAGAAGCTGT	TCCCCATGGCCATCTCG	Simon Griffiths, JIC

Supplementary Table S3. ***TB-B1*** **alleles of 8-way MAGIC lines investigated in this study.**

MEL ID	TB-B1 genotype
002-1	*TB-B1b*
002-3	*TB-B1b*
002-5A	*TB-B1a*
002-8	*TB-B1a*
004-2	*TB-B1a*
004-3	*TB-B1a*
005-7	*TB-B1a*
005-8A	*TB-B1b*
006-3	*TB-B1b*
008-1	*TB-B1a*
008-2	*TB-B1a*
008-3	*TB-B1a*
010-1b	*TB-B1a*
010-3	*TB-B1a*
010-5	*TB-B1a*
011-1b	*TB-B1b*
011-2a	*TB-B1a*
011-3	*TB-B1b*
011-4	*TB-B1a*
012-4	*TB-B1a*
014-7	*TB-B1a*
015-3	*TB-B1b*
015-8	*TB-B1a*
016-4	*TB-B1b*
017-4	*TB-B1b*
017-7	*TB-B1b*
020-3	*TB-B1a*
020-7	*TB-B1a*
023-5	*TB-B1a*
024-2	*TB-B1a*
024-5	*TB-B1a*
025-1d	*TB-B1a*
025-1e	*TB-B1a*
025-1f	*TB-B1a*
026-1b	*TB-B1b*
029-2b	*TB-B1b*
032-2	*TB-B1b*
033-5	*TB-B1a*
033-7	*TB-B1a*
034-7	*TB-B1a*
035-1	*TB-B1a*
036-7	*TB-B1a*
036-8	*TB-B1b*
043-1	*TB-B1b*
043-2	*TB-B1a*
043-3	*TB-B1a*
043-5	*TB-B1b*
043-6	*TB-B1b*

044a-1	*TB-B1b*
045-1b	*TB-B1b*
046-1	*TB-B1b*
046-2	*TB-B1b*
049-2	*TB-B1a*
052-7	*TB-B1a*
056-1	*TB-B1b*
056-4	*TB-B1a*
057-1a	*TB-B1a*
059-1b	*TB-B1a*
060-1b	*TB-B1b*
060-3	*TB-B1b*
062-2	*TB-B1a*
062-3	*TB-B1a*
064-1b	*TB-B1a*
070-1c	*TB-B1b*
071-1b	*TB-B1b*
072-1a	*TB-B1b*
072-1c	*TB-B1b*
072-1d	*TB-B1b*
077-3	*TB-B1a*
083-1	*TB-B1b*
084-3	*TB-B1b*
086-3	*TB-B1b*
086-7	*TB-B1b*
088-1	*TB-B1a*
088-3	*TB-B1a*
088-6	*TB-B1a*
089-4	*TB-B1a*
090-1a	*TB-B1a*
090-2c	*TB-B1a*
095-1	*TB-B1a*
096-6	*TB-B1a*
097-1	*TB-B1b*
097-2	*TB-B1b*
099-4	*TB-B1b*
101-7	*TB-B1a*
103-1	*TB-B1a*
104-4	*TB-B1b*
105-1	*TB-B1a*
114-8	*TB-B1b*
119-1	*TB-B1a*
119-2	*TB-B1b*
119-4	*TB-B1b*
119-5	*TB-B1b*
120-3	*TB-B1a*
122-2b	*TB-B1a*
122-4	*TB-B1a*
123-1	*TB-B1a*
126-2	*TB-B1b*
128-3	*TB-B1a*
128-4	*TB-B1a*

129-3	*TB-B1a*
129-6	*TB-B1a*
130-4	*TB-B1a*
132-4	*TB-B1a*
139-6	*TB-B1a*
139-7	*TB-B1a*
140-1b	*TB-B1a*
140-3	*TB-B1a*
145-2	*TB-B1a*
146-1b	*TB-B1a*
152-2a	*TB-B1b*
154-3	*TB-B1a*
156-4	*TB-B1b*
158-4	*TB-B1b*
158-7	*TB-B1b*
159-8	*TB-B1a*
161-2	*TB-B1a*
168-1b	*TB-B1b*
170-3	*TB-B1a*
170-8	*TB-B1a*
179-2	*TB-B1b*
182-3b	*TB-B1a*
183-1	*TB-B1a*
185-2	*TB-B1b*
186-1b	*TB-B1a*
187-2a	*TB-B1b*
189-3	*TB-B1a*
190-4	*TB-B1b*
191-4	*TB-B1a*
191-6	*TB-B1a*
196-4	*TB-B1b*
198-2	*TB-B1b*
198-3	*TB-B1b*
199-3	*TB-B1b*
201-4	*TB-B1b*
201-5	*TB-B1b*
202-2	*TB-B1a*
202-4	*TB-B1b*
203-8	*TB-B1b*
204-4	*TB-B1a*
206a-1a	*TB-B1b*
207-6	*TB-B1a*
207-7	*TB-B1a*
208-6	*TB-B1a*

MEL = MAGIC Elite Line from the 8-way winter wheat population, NIAB UK. MEL is the prefix for each line.
